# Could cone-beam computed tomography demonstrate the lateral accessory canals?

**DOI:** 10.1186/s12903-017-0430-1

**Published:** 2017-11-29

**Authors:** Yan Ji, Shanhui Wen, Shu Liu, Min Zhu, Menghuan Yao, Tiemei Wang, Zitong Lin

**Affiliations:** 1 Department of Preventive Dentistry, Nanjing Stomatological Hospital, Medical School of Nanjing University, Nanjing, China; 2 Department of Dentomaxillofacial Radiology, Nanjing Stomatological Hospital, Medical School of Nanjing University, Nanjing, China; 3 Department of Endodontics, Nanjing Stomatological Hospital, Medical School of Nanjing University, Nanjing, China

**Keywords:** Cone-beam computed tomography, Canal staining and tooth clearing technique, Mandibular incisor, Root canal morphology, Voxel size, Radiation dose, Radiography

## Abstract

**Background:**

Recently, using cone-beam computed tomography (CBCT) to assess root canal morphology has become popular; however, few studies have examined its efficiency to assess the entire root canals, including the tiny lateral and accessory canals (LACs). This study aimed to assess the ability of CBCT to evaluate the root canal of mandibular incisors at three different scanning settings, compared with the canal staining and tooth clearing (CS) technique as the gold standard.

**Methods:**

CBCT images of 70 extracted mandibular incisors were taken using NewTom VG CBCT at high-resolution scan mode (HZ), zoom scan mode (ZS), and full scan mode (FS), with different scanning settings. A radiologist, a postgraduate student, and an endodontist assessed the root canal morphology in a blinded manner. The number of root canals (NC), canal configuration according to Vertucci’s classification (VC), and LACs were evaluated twice by each evaluator using the CBCT images, in comparison with CS. Comparisons of the differences were used the chi-square test, and the intra-evaluator and inter-evaluator agreement were used the Kappa statistics; the significance level was set at 0.05.

**Results:**

The voxel dimension of HZ, ZS and FS modes were 0.125 mm, 0.20 mm and 0.25 mm respectively, and the HZ mode had significant increased scanning doses. For NC, the diagnostic accuracy was >90% in all three modes, with no significant difference among the evaluators and modes. VC and LAC could only be evaluated in HZ mode. For VC, the accuracies were 97.1%, 94.3%, and 92.9% respectively, with no significant differences among the three evaluators. For LAC, the accuracies were 80.0%, 13.3%, and 33.3% respectively, and there were significant differences among the three evaluators. Intra-evaluator agreement was excellent, with the kappa values indicating “perfect” to “substantial” agreement. Inter-evaluator agreement was excellent for NC and VC; however, Kappa values could not be analyzed due to LACs detected were so variable.

**Conclusions:**

As far as possible, the HZ mode should be chosen to demonstrate the root canal system, and partial LACs could be detected using this mode; however, the potential benefit of the diagnostic information must be weighed against the increased radiation dose.

## Background

Knowledge of the morphology of root canals is a prerequisite for successful root canal treatment (RCT), which is characterized by thorough canal debridement and effective filling of the root canal system [1]. Therefore, a precise demonstration and efficient assessment of the internal anatomy of teeth before RCT are necessary.

Various methods, such as the canal staining and tooth clearing technique, transverse cross-sectioning, ultrasound examination [2–4], and scanning electron microscopy [5] are used to assess the internal anatomy of teeth. Among these, the canal staining and tooth clearing technique is considered as the gold standard, which is capable of demonstrating the entire root canal system, including the lateral accessory canals (LACs) [1]. However, the ideal technique should be accurate, non-invasive and feasible in vivo. Cone-beam computed tomography (CBCT) is a high-resolution, non-invasive imaging technique that allows three-dimensional visualization of anatomical structures, without exposing the patient to high amounts of ionizing radiation [6]. CBCT has been used to detect complicated and varied root canal structures [7] and to study the distribution of different root canal types in populations [8, 9].

Although CBCT can help clinicians to assess root canal morphology accurately, most previous studies have focused mainly on its use for determining the number of root canals [8, 10, 11]; few studies have examined its possibility for assessing the entire root canal system, including the tiny LACs. Moreover, images with higher resolution and sharpness are required when a more detailed root canal morphology evaluation is performed, and different scanning parameters could affect image quality significantly [12, 13]. In this study, we aimed to fill the gap in the available information in this field by assessing the number of root canals, the Vertucci’s classification, and the LACs of the mandibular incisors, using CBCT at three different scanning settings in comparison with the canal staining and tooth clearing technique as the gold standard.

## Methods

### Subjects

The study was performed on a total of 70 mandibular incisors collected from the Oral and Maxillofacial Surgery Department from March 8, 2014 to September 12, 2014. Only teeth with intact roots and mature apices that had previously not undergone endodontic or restorative treatment were collected. Both central and lateral incisors were eligible for inclusion in the study.

The teeth were washed under tap water immediately after extraction, and any remaining periodontal tissue or calculus was removed by scaling. They were stored in 0.1% thymol solution until the sample collection was complete.

### CBCT

The CBCT images were taken using a NewTom VG scanner (QR srl, Verona, Italy) according to the manufacturer’s recommended protocol. The teeth were arranged in sequence for identification and location on a piece of polystyrene foam and then scanned in three different modes: high-resolution zoom scan (HZ) mode, zoom scan (ZS) mode and full scan (FS) mode, the scanning parameters of the three modes are showed in Table 1. The CBCT images were viewed on a 29.7-in. screen MX300W LCD monitor (EizoNanao Corporation, Japan) and analyzed with the inbuilt software NNT 5.5(QR srl, Verona, Italy). Axial, sagittal and coronal images at different root levels were displayed on the monitor. Multiplanar reconstruction (MPR) was used for the evaluation. We selected the thinnest thickness which could be selected in the three different scanning modes to reconstruct the optimal images for root canal evaluation: 0.125 mm in HZ mode, 0.2 mm in ZS mode and 0.25 mm in FS mode. The windows width and level were set manually using the image-processing tool of the software to ensure optimal visualization.

**Table 1:** Scanning parameters in three different modes

Scanning parameters	HZ	ZS	FS
Field of view(cm)	12 × 8	12 × 8	15 × 15
Voxel size(mm)	0.125	0.20	0.25
Kilovolt (kV)	110	110	110
Milliampere second (mAs)	35.19	4.59	3.07
Exposure time(s)	5.4	3.6	3.6
Original image number	540	360	360

### Canal staining and clearing technique

After CBCT scanning, the canal staining and clearing technique was performed for all the 70 mandibular incisors [2]. Endodontic accesses were prepared for all the samples. The pulp tissue was removed with a barbed broach, and the teeth were immersed in 3.25% sodium hypochlorite overnight to remove adherent soft tissues. Then, the teeth were rinsed in running tap water for 2 h and dried overnight. Subsequently, the teeth were placed in 20-ml injectors containing India ink. The injectors were pulled to lower the pressure to within subatmospheric levels, until the ink penetrated the root canal system. The teeth were then demineralized by immersion in 5% nitric acid for 6 days at room temperature; the acid solution was changed every 24 h. After washing under running tap water for 4 h, the teeth were dried and dehydrated using ascending concentrations of ethyl alcohol (70%, 96% and 99%) for 12 h each. Finally, the dehydrated teeth were placed in methyl salicylate, which rendered them transparent.

### Evaluation of root canal morphology

**Fig. 1:**
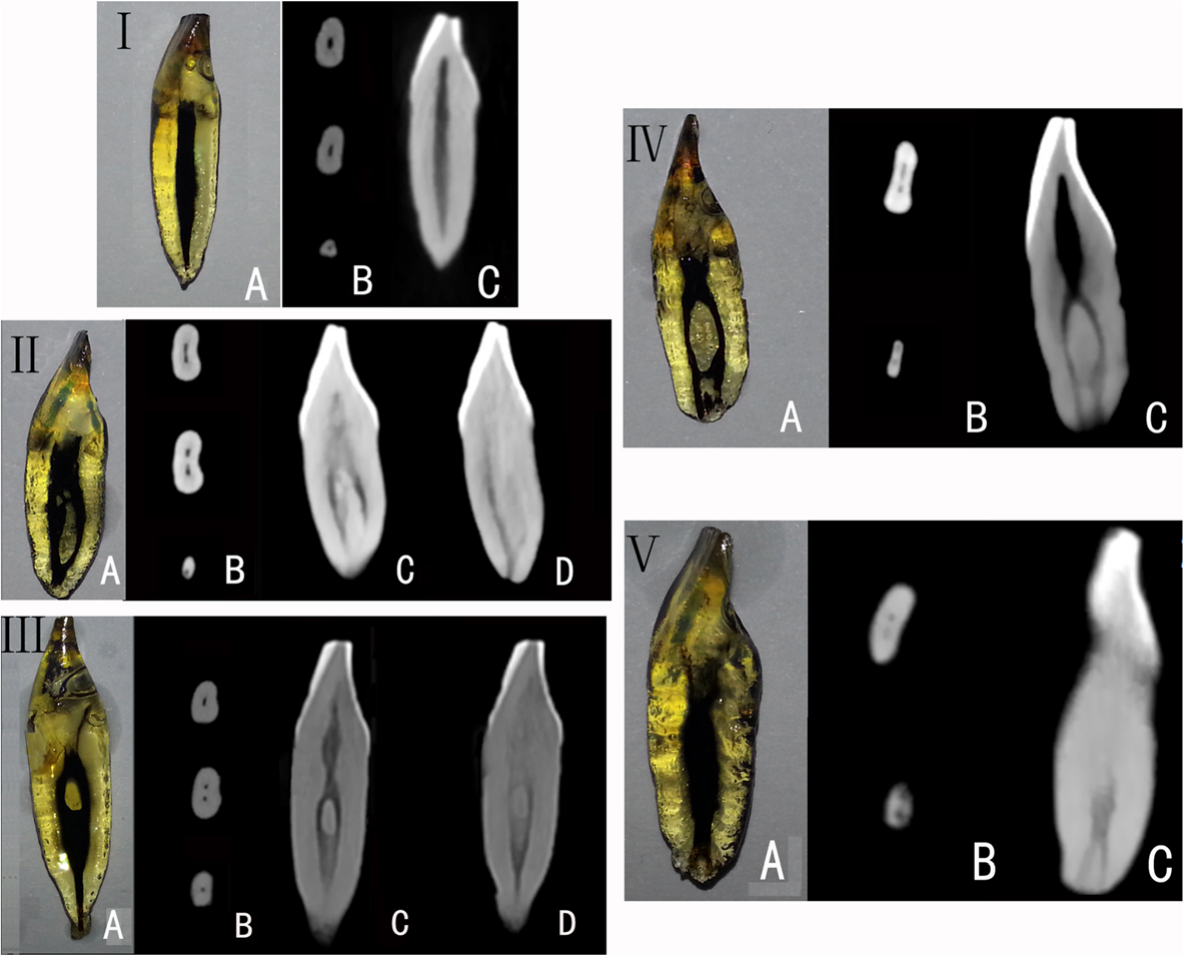
Canal configuration according to Vertucci’s classification: type I, II, III, IV and V. **a** Teeth with the canal staining and tooth clearing technique demonstrating root canal configuration. **b**-**d** CBCT images in HZ mode: (**b**) axial images; (**c**) and (**d**) reconstructed sagittal images

**Fig. 2:**
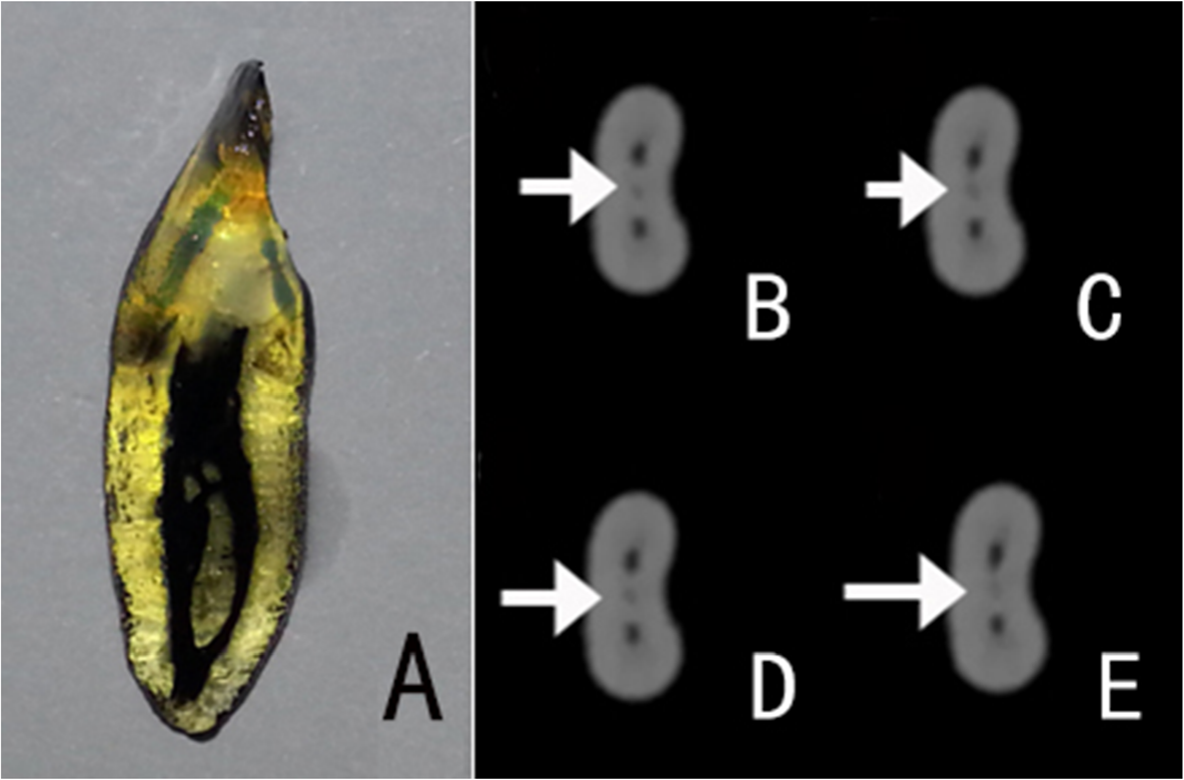
Image of a tooth with inter-canal communications. The white arrows denote the inter-canal communication. **a** Cleared tooth demonstrating inter-canal communications. **b**–**e** Continuous CBCT images in the axial view: A tiny low-density canal can be seen migrating between the two canals

**Fig. 3:**
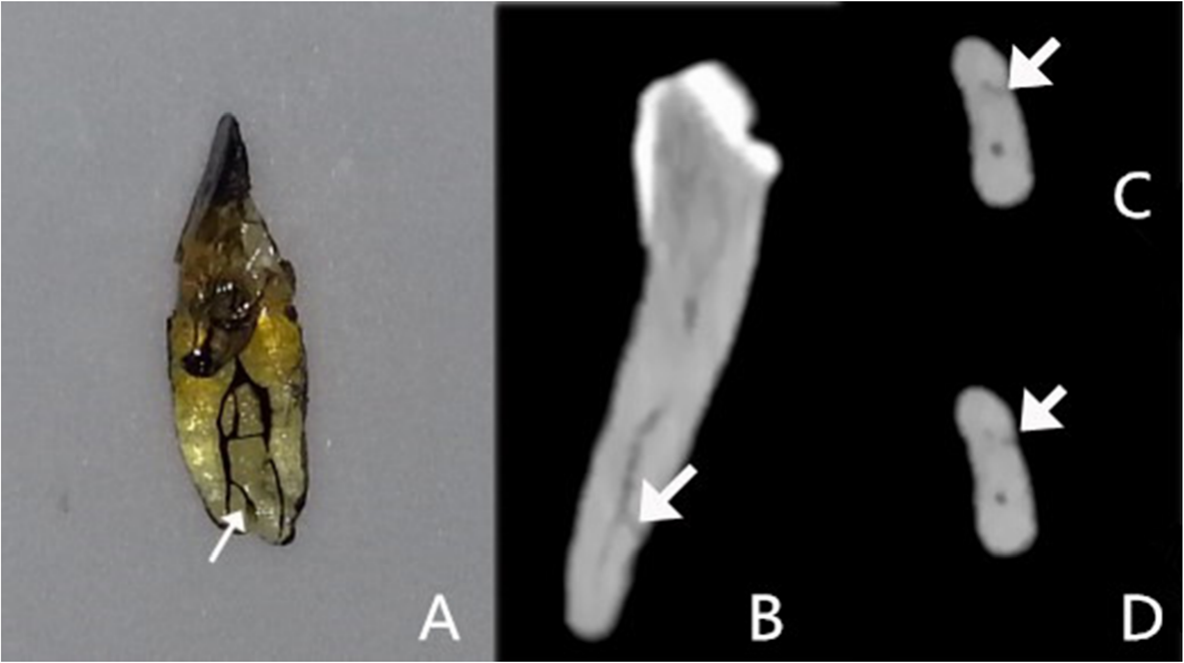
Image of a tooth with lateral canals. The white arrows denote the lateral canal. **a** Cleared tooth demonstrating the lateral canal. **b** CBCT image with oblique reconstruction. **c** and **d** CBCT images in the axial view: A subtle branch can be seen extending from the labial canal to the root surface

**Fig. 4:**
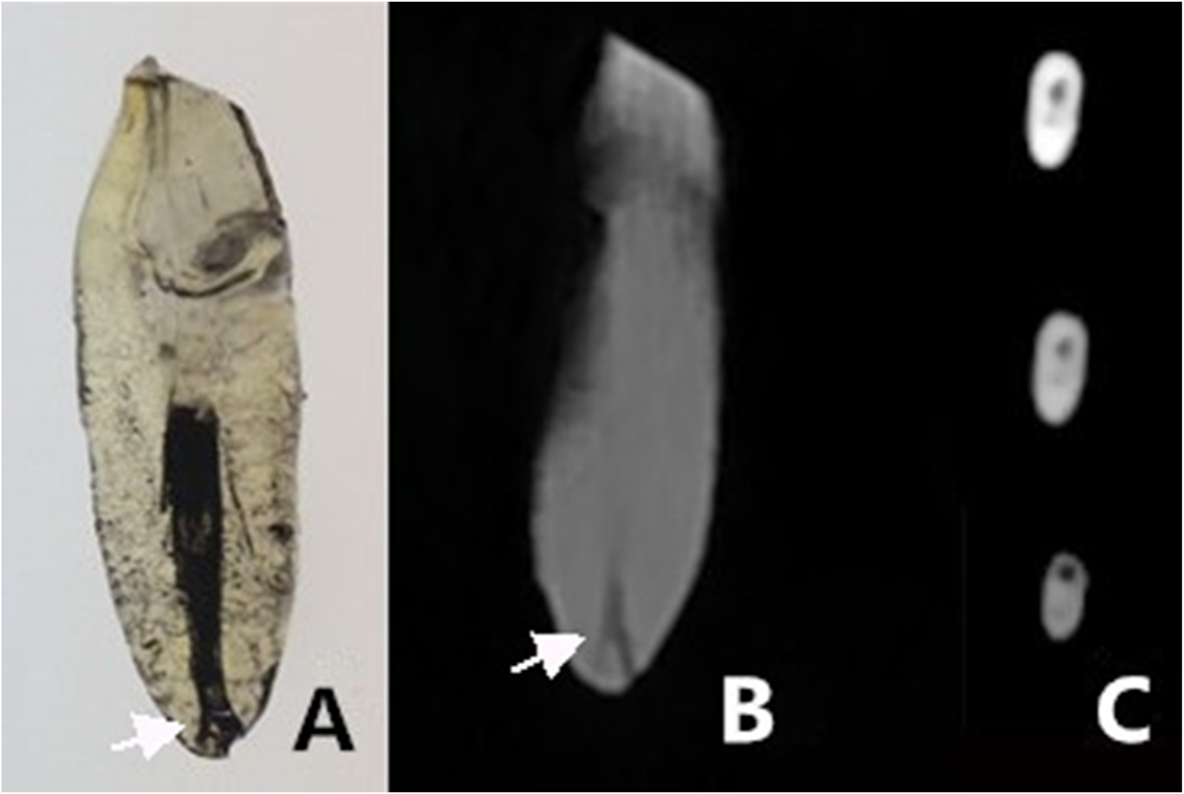
Image of a tooth with apical deltas. **a** Cleared tooth demonstrating the apical delta. **b** CBCT image with MPR reconstruction. **c** CBCT images in the axial view. The main root canal can be seen migrating to the lingual side of the root. A subtle branch can be seen extending from the main root canal to the labial side of the root

The following observations were made for all the specimens:

1. The number of root canals (single canal or two canals); the presence of two canals was assumed if two canals were identified in any of three continuous axial slices from the pulp chamber to the apex.
2. Canal configuration was classified using the Vertucci’s classification [1] as

- type I: a single canal present from the pulp chamber to the apex
- type II: two separate canals leave the pulp chamber, but later join to form one canal to the site of exit
- type III: one canal leaves the pulp chamber, divides into two within the root, and then merges to exit as one canal
- type IV: two separate and distinct canals present from the pulp chamber to the apex
- type V: a single canal leaves the pulp chamber but divides into two separate canals, with two separate apical foramina (Fig. 1).

3) presence of lateral accessory canals [1]

- intercanal communications (Fig. 2)
- lateral canals (Fig. 3)
- apical deltas (Fig. 4)

**Fig. 5:**
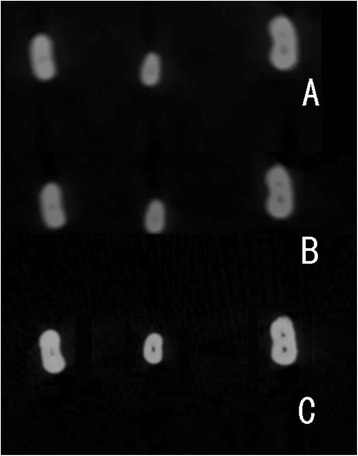
Example of CBCT images of the same incisor near to the apical region, (**a**) was in FS mode, (**b**) was in ZS mode and (**c**) was in HZ mode. The root canal morphology in HZ mode is much sharper than the other modes

The root canal morphology of some specimens near the apical regions were blurred when displayed in the ZS and FS modes (Fig. 5); therefore, Vertucci’s classification was only evaluated in the HZ mode; and LACs could not be detected in the ZS and FS modes at all. A radiologist (with 5 years of experience as an attending doctor), a postgraduate student in oral and maxillofacial radiology (with 3 months of training), and an endodontist (with 5 years of experience as an attending doctor) evaluated the root canal morphology on the CBCT images in a blinded manner. Before the evaluation, all the three examiners have calibration procedure. The calibration consisted of the identification of number of root canal, Vertucci’s classification of mandibular incisors of 30 patients’ CBCT images and learning of previous found lateral accessory canals in our CBCT database. The evaluation was performed again after 3 months by all three evaluators. After that, all three evaluators together evaluated the root canal morphology of all the 70 transparent specimens manually, and the result was used as the gold standard to compare with the results obtained by CBCT images. All the evaluation was performed in the same diagnosis room with normal brightness.

### Statistical analysis

The accuracy of the root canal morphology was calculated as the percentage of correct findings of the first time of evaluation with the CS technique findings as the reference. For the number of root canals, the accuracy of each evaluator in the three modes and the accuracy of the three evaluators in each mode were compared using a chi-square test. For Vertucci’s classification and the LACs identified in the HZ mode, the accuracy of the three evaluators was also compared using the chi-square test. *p* < 0.05 was set as the significance level.

For all the above observations, inter-examiner agreement was analyzed among the three evaluators and intra-evaluator agreement was analyzed between the first and second evaluation. Interpretation of the Kappa statistics (κ) was quoted from the guidelines of Landis and Koch: less than 0.00 poor agreements, 0.00–0.20 slight agreement, 0.21–0.40 fair agreement, 0.41–0.60 moderate agreement, 0.61–0.80 substantial agreement and 0.81–1 almost perfect agreement [14]. SPSS version 11.5 was used (SPSS Inc., Chicago, IL, USA) for the statistical analysis.

## Results

Table 1 provided the scanning parameters of the FS, ZS, and HZ modes of NewTom CBCT. The original image number corresponds to exposure time and the milliampere second (mAs) was the product of milliampere and exposure time [13]. Therefore, the differences among the three modes actually existed at: field of view, voxel size and radiographic exposure related parameters (KV, mA and exposure time). The field of view of the HZ and ZS modes were both small, and the FS was medium [13]. The kilovolt value of the three modes was the same.

The accuracy of assessment of the number of root canals in the FS, ZS, and HZ modes was >90% for all three evaluators (Table 2); the differences between the three evaluators and between the three modes were not statistically significant (*p* > 0.05).

**Table 2:** Accuracy of NC as assessed by three evaluators in the FS, ZS and HZ modes

Evaluator	Accuracy			*P*
	FS	ZS	HZ	
Radiologist	91.4%	91.4%	97.1%	0.811 ^a^
Student	90.0%	90.0%	95.7%	0.693 ^a^
Endodontist	90.0%	91.4%	97.1%	0.747 ^a^
*p*	0.976^a^	0.811 ^a^	0.978 ^a^	

*HZ* high-resolution zoom scan mode, *ZS* zoom scan mode, *FS* full scan mode^,a^ *p > 0.05*

Vertucci’s classification and LACs were only evaluated in the HZ mode. In the HZ mode, for Vertucci’s classification, the accuracies of the radiologist, the postgraduate student, and the endodontist were comparable (*p* = 0.930); however, for evaluation of LACs, there were significant differences between the three evaluators (*p* = 0.012). (Table 3).

**Table 3:** Accuracy of Vertucci’s classification and lateral accessory canal as assessed by the three evaluators in HZ mode

Evaluator	Accuracy	
	VC	LAC
Radiologist	97.1%	80.0%
Student	94.3%	13.3%
Endodontist	92.9%	33.3%
*p*	0.930^b^	0.012^a^

*VC* canal configuration was classified using Vertucci’s classification, *LAC* lateral accessory canal, ^a^ *p < 0.05;* ^b^ *p > 0.05*

Table 4 shows inter-evaluator agreement of the three examiners. The kappa values were mostly between 0.81 and 1, indicating “perfect” agreement for NC in three different modes and VC in the HZ mode; however, due to LACs detected by the student and the endodontist were too few, the kappa values could not be calculated.

**Table 4:** Inter-examiner agreement of the NC as assessed by the three evaluators in the FS, ZS and HZ modes; VC and LAC as assessed by the three evaluators in HZ mode

Evaluator	*κ*				
	FS(NC)	ZS(NC)	HZ(NC)	HZ(VC)	HZ (LAC)
Radiologist vs. Student	0.856	0.964	0.966	0.941	/
Radiologist vs. Endodontist	0.851	0.928	0.935	0.909	/
Student vs. Endodontist	0.930	0.890	0.902	0.845	/

*HZ* high-resolution zoom scan mode, *ZS* zoom scan mode, *FS* full scan mode

*NC* number of root canals, *VC* canal configuration classified using Vertucci’s classification, *LAC* lateral accessory canal

Table 5 shows the intra-evaluator agreement for all the observations. The kappa values were mostly between 0.81 and 1, indicating “perfect” agreement between the first and the second evaluations; the exception was for LACs assessment in the HZ mode, where the postgraduate student and the endodontist had kappa values of 0.680 and 0.641, respectively, indicating “substantial” agreement.

**Table 5:** Intra-examiner agreement of the NC as assessed by the three evaluators in the FS, ZS and HZ modes; VC and LAC as assessed by the three evaluators in HZ mode

Evaluator	*κ*				
	FS(NC)	ZS(NC)	HZ(NC)	HZ(VC)	HZ (LAC)
Radiologist	0.963	0.964	0.967	0.915	0.887
Student	0.963	0.897	0.967	0.885	0.680
Endodontist	0.932	0.963	1	0.910	0.641

*HZ* high-resolution zoom scan mode, *ZS* zoom scan mode, *FS* full scan mode

*NC* number of root canals, *VC* canal configuration classified using Vertucci’s classification, *LAC* lateral accessory canal

## Discussion

In the present study, the accuracy of CBCT to identify the number of root canals and LACs, and to enable Vertucci’s classification, were evaluated, using the canal staining and tooth clearing technique as the gold standard for comparison. The study was performed on mandibular incisors because they are the smallest permanent teeth, with rather tiny root canals and a relatively complex and variable root canal configuration [2, 15, 16].

To evaluate the number of root canals, the accuracy of assessment in the three different modes ranged from 90.0% to 97.1%. These results are consistent with those of Neelakantan et al., who found high agreement between CBCT and a modified canal staining and clearing technique to identify the number of root canals in 95 extracted teeth [10]. We found no significant difference between the HZ, ZS, and FS modes. Inter- and intra-evaluator agreement was also excellent. These findings indicate that CBCT is a reliable method for assessing the number of root canals. Therefore, in the clinic, if it is only necessary to know how many root canals are present, CBCT, in any one of the three modes, could be used.

In the present study, the Vertucci classification of root canal configuration and assessment of LACs were only performed in the HZ mode, because the configuration of the root canal nearing the apical foramen region was not clearly demonstrated in the ZS and FS modes (Fig. 5). Zhang et al. [17] have also reported poor demonstration of the apical area root canal systems of the first molar on CBCT images, and the voxel size used in his study was 0.5 mm, which was larger than that used here. In the present study, no LACs were detected in the ZS and FS modes, probably because the LACs are very small. Miyashita et al. [15] reported that more than 80% of LACs have a thickness ≤ 0.15 mm.

The differences parameters of the HZ, ZS and FS modes included field of view, voxel sizes, and radiographic exposure related parameters (KV, mA and exposure time). The field of view would not influence image quality; however, the voxel size and the radiographic exposure factors do have an influence. In this study, the HZ mode had a voxel size of 0.125 mm, a 1.5 times exposure time, and significant increased mAs. Previous studies focused on the influence of different voxel sizes on the accuracy of root canal morphology evaluation [12, 18]. In Bauman’s research on mesiobuccal canals in the maxillary molars, in which voxel sizes of 0.40, 0.30, 0.20 and 0.125 mm were used, the detection rate increased from 60.1% at 0.4 mm to 93.3% at 0.125 mm. Significant differences were observed between the different voxel sizes, but not between the 0.2 and 0.125 mm voxel sizes [12], which indicated that the effect of increasing voxel dimension would decline when the voxel dimension was small enough. Celikten et al. evaluated shaping ability of two nickel-titanium rotary systems using cone beam computed tomography with voxel sizes of 0.125 mm and 0.100 mm, and the results also showed the two voxel resolutions had similar evaluation values [19]. And our study showed similar results; there were no significant differences among the three modes in detecting the number of root canals.

The Vertucci’s classification requires precise demonstration of the root canal from the pulp chamber to the apex, and LACs are quite tiny and subtle. In this study, the root canal near to the apical regions was ambiguous and no LACs were detected in the FS and ZS modes. It seems that decreased voxel size and increased mAs together improved the quality of CBCT image and made the precise evaluation of root canal system possible. However, the NEWTOM CBCT only has three fixed scanning settings; therefore, further research to separate the influence of voxel size and mAs is needed in future. Moreover, a significant increased mAs was found at the HZ mode compared; therefore, this should be taken into account when the HZ mode is chosen in clinical practice. It is critical for healthcare providers to weigh the potential benefit of diagnostic information against the risk of the imaging procedure [20]. Additionally, we should take into account that our study was just an in vitro study, the image motion artefacts could be completely avoided [21], and this also increased the detection of LACs.

It has been reported that the diagnostic accuracy of second-year trainees is significantly higher than that of first-year trainees and endodontic staff with regard to mesiobuccal canal (MB) detection (87.9% vs. 77.1% and 76.8% respectively. Intra-rater agreement increased with higher resolution scans (41.1% to 96.4%) [12]. In this study, the detection rate of lateral accessory canals by the senior radiologist in HZ mode was much higher than that for the other two evaluators, which showed that professional training could improve the accuracy of diagnosis, especially when performing a precise evaluation of subtle structure of the root canal system. In our study, LACs presented with the following features: tiny round or elliptical low density shadow and gradually move to the root surface (Figs. 2, 3 and 4); the movement is an important image feature in CBCT images.

## Conclusions

The present study showed that CBCT, in any of three modes (HZ, ZS, and FS), could be used to evaluate the number of root canals. However, the ZS and FS modes are inadequate for Vertucci’s classification of canal configuration or for identification of lateral accessory canals. CBCT in the HZ mode demonstrated optimum image quality and is the best choice to evaluate the root canal system; however, the increased radiation dose should also be considered in clinic practice. Furthermore, diagnostic accuracy, especially to identify subtle lateral accessory canals, improves with professional training.

## Abbreviations

CBCT: Cone-beam computed tomography
CS: Canal staining and tooth clearing technique
FS: Full scan mode
HZ: High-resolution zoom scan mode
LAC: Lateral accessory canal
NC: Number of root canals
VC: Canal configuration classified using Vertucci’s classification
ZS: Zoom scan mode
